# Characterization of the PAS domain in the sensor-kinase BvgS: mechanical role in signal transmission

**DOI:** 10.1186/1471-2180-13-172

**Published:** 2013-07-24

**Authors:** Elian Dupré, Alexandre Wohlkonig, Julien Herrou, Camille Locht, Françoise Jacob-Dubuisson, Rudy Antoine

**Affiliations:** 1 Institut Pasteur de Lille, Center for Infection and Immunity, Lille, France; 2 CNRS UMR8204, Lille, France; 3 INSERM U1019, Lille, France; 4 Univ Lille Nord de France, 59019 Lille, France; 5 VIB Department of Structural Biology, Free University of Brussels, Pleinlaan 2, Brussels 1050 Belgium; 6 Present address: Department of Biochemistry and Molecular Biology, University of Chicago, Chicago, IL, USA

**Keywords:** Two-component system, Bordetella, Virulence regulation, PAS domain, Signaling

## Abstract

**Background:**

In bacteria, signal-transduction two-component systems are major players for adaptation to environmental stimuli. The perception of a chemical or physical signal by a sensor-kinase triggers its autophosphorylation. The phosphoryl group is then transferred to the cognate response regulator, which mediates the appropriate adaptive response. Virulence of the whooping cough agent *Bordetella pertussis* is controlled by the two-component system BvgAS. Atypically, the sensor-kinase BvgS is active without specific stimuli at 37°C in laboratory conditions and is inactivated by the addition of negative chemical modulators. The structure of BvgS is complex, with two tandem periplasmic Venus flytrap domains and a cytoplasmic PAS domain that precedes the kinase domain, which is followed by additional phosphotransfer domains. PAS domains are small, ubiquitous sensing or regulatory domains. The function of the PAS domain in BvgS remains unknown.

**Results:**

We showed that recombinant BvgS PAS proteins form dimers that are stabilized by α helical regions flanking the PAS core. A structural model of the PAS domain dimer was built and probed by site-directed mutagenesis and by biochemical and functional analyses. Although we found no ligands for the PAS domain cavity, its integrity is required for signaling. We also showed that the structural stability of the PAS core and its proper coupling to its flanking N- and C-terminal α helices are crucial for BvgS activity.

**Conclusions:**

We propose that a major function of the BvgS PAS domain is to maintain conformational signals arising from mechanical strain generated by the periplasmic domain. The tight structure of the PAS core and its connections with the upstream and downstream helices ensure signaling to the kinase domain, which determines BvgS activity. Many mild substitutions that map to the PAS domain keep BvgS active but make it unresponsive to negative modulators, supporting that modulation increases conformational strain in the protein.

## Background

Two-component systems (TCS) are one of the predominant signal transduction systems in bacteria, which are often essential to enable microorganisms to adapt to changes of their environment [1]. They regulate important developmental programs as well as bacterial virulence in response to environmental stimuli. Typically, they are composed of a transmembrane sensor-kinase protein and a cytoplasmic response regulator. Perception of a chemical or physical signal by the sensor leads to autophosphorylation, and then transfer of the phosphoryl group to the response regulator [2]. Thus activated, the latter mediates a specific, frequently transcriptional, cellular response.

The whooping cough agent *Bordetella pertussis* colonizes the upper respiratory tract of humans. Its virulence regulon is controlled by the TCS BvgAS. At 37°C and in laboratory growth conditions, the BvgAS system is activated, leading to the transcription of a number of genes coding for virulence factors, necessary for infection [3]. In contrast to most two-component sensor-kinases, BvgS appears to be active in its basal state. Switching to the avirulent Bvg^-^ phase can be triggered by the addition of chemical modulators, such as nicotinate or sulfate ions. However, the authentic negative modulators that might act at specific stage(s) of the bacterium’s life cycle remain to be identified. BvgS is a hybrid sensor-kinase harboring several cytoplasmic domains that mediate a complex phospho-transfer cascade [4]. It also contains three potential perception domains, two periplasmic Venus flytrap (VFT) domains in tandem and a cytoplasmic Per/ArnT/Sim (PAS) domain followed by the kinase domain [5]. We have established that the second VFT domain, VFT2, binds nicotinate and related negative modulator molecules [6]. BvgS is the prototype for VFT-containing sensor-kinases mostly found in Proteobacteria whose molecular mechanisms are poorly understood.

In this work, we characterized the PAS domain of BvgS (PAS_Bvg_). PAS domains are structurally conserved, 100- to 120-residue-long signaling modules with sensory and regulatory functions, present in kinases, chemoreceptors and other types of proteins in all branches of the phylogenetic tree [7,8]. They are composed of a central, five-stranded anti-parallel β sheet flanked by α helices. Many PAS domains appear to form dimers *in vitro* and *in vivo*[8]. A subset of PAS domains harbors heme, flavine nucleotide or other cofactors for perception of physical parameters such as light or O_2_[9]. Some cytoplasmic PAS domains appear to modulate signal transmission rather than to directly perceive a signal [8,10,11]. Finally, some PAS domains, including the periplasmic ‘PDC’ (PhoP/DcuS/CitA) domains found in many bacterial TCS sensor-kinases bind small chemical ligands, which triggers signal transduction [12-15]. Although the presence of a PAS domain in BvgS has been recognized for over 20 years [16,17], its role is still unknown. Here, we show that this domain is required for transmission of signals from the periplasm.

## Methods

### Strains and plasmids

The sequence coding for the PAS core domain was amplified by PCR using the PAScore UP and PAScore LO oligonucleotides as primers (see Additional file 1: Table S1). The amplicon was inserted in pCRII-TOPO (Invitrogen) and sequenced. It was then introduced as a BamHI-HindIII fragment into the corresponding sites of pQE-30 (Qiagen). The resulting plasmid encodes the PAS_Bvg_ core with an N-terminal His tag. Next, two longer constructs were prepared using the primers PAS His UP and PAS His LO and PAS GB1 UP and PAS GB1 LO. The first amplicon was introduced into pQE30 as a BglII-HindIII fragment, and the other was introduced into pGEV2 [18] as a BamHI-XhoI fragment. The first plasmid codes for PAS_Bvg_ flanked by its N- and C-terminal helices and with an N-terminal 6-His tag. The second codes for a fusion between the GB1 domain and the same BvgS fragment. Finally, sequences coding for PAS_Bvg_ recombinant proteins of various lengths were amplified by PCR using a combination of the following primers: PAS N1UP, PAS N2UP or PAS N3UP and PAS C1LO, PAS C2LO or PAS C3LO (Additional file 1: Table S1). The amplicons were restricted as *Bsa*I fragments, introduced into the corresponding sites of the pASK-IBA35+ vector (IBA) and sequenced. The resulting plasmids encode recombinant PAS_Bvg_ proteins with an N-terminal His tag in all cases.

Selected residues were replaced by site-directed mutagenesis as described in [19]. Briefly, the Bvg-BglII and Bvg-Xba primers were used with the ‘LO’ and ‘UP’ primers of each pair of mutagenic oligonucleotides to perform overlapping PCRs (Additional file 1: Table S1; the names of the mutagenic oligonucleotides relate to the corresponding substitutions). After verification of the sequences, the mutated fragments were exchanged for their wild type (wt) counterparts in a plasmid that contains most of the *bvgAS* operon in tandem restriction cassettes [19]. The *bvgS* sequence coded by that plasmid corresponds to that of *Tohama* I BP1877, except that a Glu codon is found at position 705, as found in most other *B. pertussis* strains [19]. The mutations were then introduced into the chromosome of BPSM_*∆bvgAS*_, a *Tohama* I derivative harboring a large deletion in the *bvgAS* operon, by using allelic exchange as described [19]. Finally, a *ptx-lacZ* transcriptional fusion was generated in each of the recombinant strains using pFUS2 [20].

The virulent BPSM_E705_ strain (wt control) and the avirulent *B. pertussis* BPSM*ΔbvgS* were described in [19]. BPSM*ΔbvgA* harbour a chromosomal deletion of *bvgA*. It was constructed by allelic replacement using homologous recombination as follows. DNA fragments flanking the *bvgA* gene were amplified from the BPSM chromosome using the pairs of oligonucleotides BvgA-UP1 and BvgA-LO1, and BvgA-UP2 and BvgA-LO2, respectively. The amplicons were used as templates for an overlapping PCR, and the resulting amplicon was introduced as an XbaI-HindIII restriction fragment into pSS1129 restricted with the same enzymes [21]. The resulting suicide plasmid was used for allelic replacement as described [21].

To introduce the substitutions of interest into the recombinant protein, the N2C3 UP and N2C3 LO primers were used to amplify the relevant gene portion from the mutagenized plasmids described above. The amplicons were then introduced into pASK-IBA35+ in the same manner as for the wt gene fragment.

### Protein production and purification

Productions of the PAS_BvgS_ core from the pQE and pGEV derivatives were performed in *Escherichia coli* SG13009(pREP4) (Qiagen) and BL21(DE3), respectively. pREP4 harbors a *lacI*^*Q*^ gene for repression of the *lac* promoter prior to induction with IPTG.

A number of conditions were tested to optimize protein production, by varying the temperature of the cultures, the absorbance at 600 nm of the culture at the time of induction, the concentration of inducer and the duration of the induction. Production of the 9 recombinant proteins from the pIBA derivatives was performed in *E. coli* BL21 (DE3). A number of inductions conditions were also tested, and the following one was identified as the most suitable. A 50-ml overnight culture in LB medium supplemented with 150 μg/ml ampicillin (LB-Amp_100_) was used to inoculate 1 liter of LB-Amp_150_ to an OD_600_ of 0.05. The culture was incubated at 37°C in a rotary shaker at 220 rpm. Expression of recombinant PAS_Bvg_ was induced at an OD_600_ of 0.4 by the addition of 200 μg/L anhydrotetracycline (IBA). After 5 h of incubation under the same conditions, the cells were harvested by centrifugation at 8,000 × g for 20 min at 4°C. Hemin (Sigma) or 5-aminolevulinic acid (Sigma) were added at a concentration of 10 mM one hour before induction in the relevant cultures. For N2C3 production at 16°C, the cultures were grown at 37°C until they reached an OD_600_ of 0.4, then switched to 16°C 30 min before addition of the inducer. Induction was performed for 16 hours.

In all cases, the cell pellets were resuspended in 10 mM Tris–HCl (pH 7.5), 150 mM NaCl, 10 mM imidazole (binding buffer) with 5 μg/ml of DNase I (Sigma) and EDTA-free protease inhibitor cocktail (Roche). Cells were disrupted by three passages in a French pressure cell, and the bacterial debris was removed by centrifugation for 20 min at 10,000 × *g*. The supernatant was loaded onto a Ni^2+^-Sepharose affinity column (GE Life Sciences) pre-equilibrated with the binding buffer. Two washing steps were performed by using successively 10 mM and 50 mM of imidazole in the binding buffer, followed by an elution step with 200 mM imidazole. The protein was further purified by gel filtration in 10 mM Tris–HCl (pH 7.5), 150 mM NaCl through a HiLoad 16/60 Superdex 75 column (GE Healthcare). All purification steps were carried out at 4°C or 12°C.

### Protein analyses

Mass spectrometry analyses were performed on an ESI-Q-TOF spectrometer (Waters, Micromass) in positive ion mode by GIGA Proteomics at the University of Liège, Belgium. Purified N2C3 was used at a concentration of 10 μM in 27 mM ammonium acetate for native conditions, or in 31.25 mM ammonium acetate, 30% acetonitrile and 0.5% formic acid for denaturing conditions.

The delipidation treatment of the purified protein was performed as described in [22]. The protein solution (4 mg/ml) was incubated with 1 ml of LIPIDEX 1000 matrix (Perkin Elmer) previously equilibrated in the gel filtration buffer, for 1 hour at 37°C under gentle agitation. The mixture was centrifuged, and the supernatant was collected and applied to the same amount of fresh LIPIDEX 1000 matrix. The incubation step was performed 6 times in total. Thermal denaturation was performed in 96-wells plate with 15 μl per well of a 30 μM protein solution and 4 × NanoOrange® (Invitrogen) diluted 125 folds from a 500 × stock solution [23]. The plates were heated from 25°C to 85°C with a ramp rate of 0.07°C/s and read by a thermocycler (LightCycler 480 II, Roche) using excitation and emission wavelengths of 465 nm and 510 nm, respectively. The Tms were determined using the LightCycler480 Software. The experiments were performed two or three times at least in triplicate. The statistical analyses were performed using the unpaired t test of the Graphpad PRISM software.

For the crystallogenesis attempts, purified PAS_Bvg_ proteins (N2C2, N2C3, N3C2 and N3C3) were concentrated using a centrifugal filter unit (Ultracel® 10 kDa membrane, Amicon® Ultra, Millipore, Billerica, MA). Crystallization screening was carried out using the sitting-drop, vapor-diffusion technique in 96-well microplates. Trays were set using a Phenix crystallization robot (Art Robbins instrument) and commercial crystallization kits (HR-Index, HR-AMSO4, HR-Cryst1&2, HR-Cryo from Hampton Research, Nextal-JCSG + from QIAGEN, Proplex and PACT from Molecular Dimensions). The drops were set up by mixing equal volumes (0.1 μl) of the protein and the precipitant solutions equilibrated against 75 μl of the precipitant solution. The protein concentrations ranged from 10 to 80 mg/ml for PAS_Bvg_ N2C3 and N2C2 and from 10 to 30 mg/ml for PAS_Bvg_ N3C2 and N3C3.

To prepare the membrane fractions of the various *B. pertussis* strains, the bacteria were grown in modified Stainer-Scholte medium (SS) [24] containing 100 μg/ml streptomycin and 10 μg/ml gentamycin. After 24 h at 37°C under rotating agitation (220 rpm) cells were harvested by centrifugation, resuspended in phosphate-buffered saline (PBS) to an OD_600_ of 5 and broken using a Hybaid Ribolyser apparatus (30 s at speed 6 in tubes containing 0.1 mm silica spheres as the lysing matrix). The lysates were clarified by centrifugation (8000 × *g*, 10 minutes), and the membrane fractions were pelleted from 1 ml of supernatants by ultracentrifugation (90 000 × *g*, 1 hour). The pellets were resuspended in 100 μl PBS and used for denaturing electrophoresis in 4-8% gradient polyacrylamide gels (Novex, Life Technologies). The proteins were then transferred electrophoretically to nitrocellulose membranes for immunoblotting. Polyclonal antibodies against BvgS were raised in rats (Eurogentec, Belgium) and used at a 1:500 dilution in PBS + 0.1% Tween 20. The secondary antibody was an anti-rat immunoglobulin- alkaline phosphatase conjugate (Promega) at a 1:7,500 dilution in the same buffer. Revelation of the blots was performed using the BCIP/NBT Color Development Substrate (Promega).

### Homology modeling

A similarity search using PSI-BLAST [25] was performed to find suitable templates. Modeller 9v8 [26] was used to build a model of the structure of the PAS domain of BvgS based on 3BWL. The protein side-chain conformations were predicted using SCWRL4 [27]. The quality of the model was assessed using PROSA II [28]. Molecular structure inspections and illustrations were made using PyMOL (PyMOL Molecular Graphics System, version 1.3, Schrödinger).

### β-galactosidase activities

The various *B. pertussis* strains harboring specific mutations in *bvgS* and a *ptx-lacZ* fusion were grown in modified SS medium containing 100 μg/ml streptomycin and 10 μg/ml gentamycin. After 24 h at 37°C under rotating agitation as above, the bacterial suspension was used to initiate cultures in 10 ml of medium either not supplemented or containing the desired concentration of modulators. The inoculation volume was adapted to compensate for slower growth in the presence of high concentrations of nicotinate. The bacteria were grown until the cultures reached an OD_600_ of 1.5, harvested by centrifugation, resuspended in Z buffer (60 mM Na_2_HPO_4_, 40 mM NaH_2_PO_4_, 10 mM KCl, 1 mM MgSO_4_ and 2,7 ml of 2-mercaptoethanol per liter added immediately before use) to an OD_600_ of 5 and broken as described above. ß-galactosidase activities were determined as described [19]. The experiments were performed in triplicate, and statistical analyses were conducted as above. The *ptx* operon codes for pertussis toxin, a virulence factor whose expression is positively regulated by BvgAS. The *ptx-lacZ* transcriptional fusion interrupts the first gene of the operon and places *lacZ* under the control of the Bvg-regulated *ptx* promoter. Thus, the levels of β galactosidase activity measured after growth in virulent, Bvg^+^ conditions reflect the activity of BvgS, while those under modulating conditions reflect the ability of BvgS to respond to the negative modulators.

## Results

### Production of recombinant PAS proteins

Among the hundreds of predicted VFT sensor-kinases many, including BvgS, harbor in their cytoplasmic moiety PAS, GAF, receiver or Hpt domains in addition to the His-kinase module [5]. When present, the PAS domain most frequently precedes the kinase domain.

**Figure 1:**
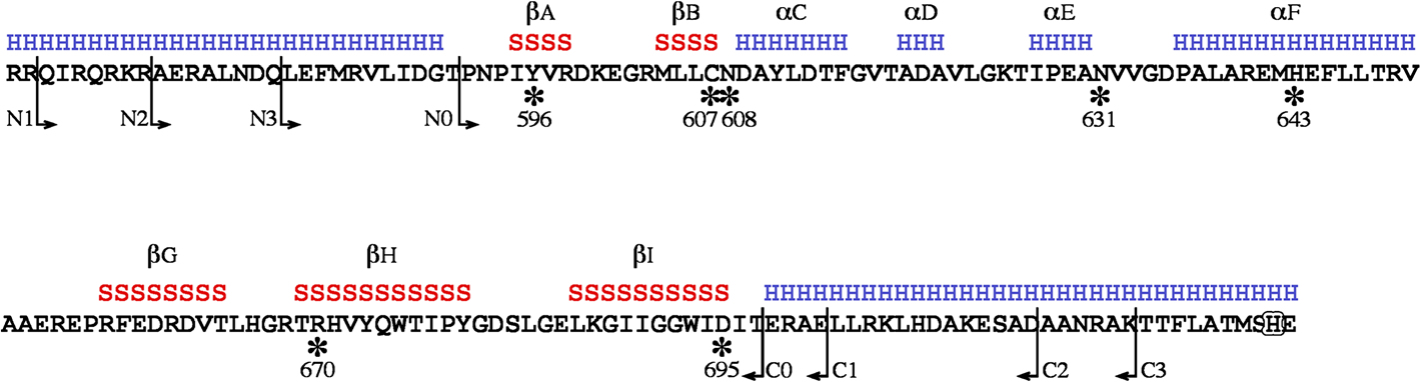
**Sequence of PAS**_**Bvg **_**and flanking regions and of the recombinant proteins produced in this work.** The predicted secondary structures are shown above the sequence, with H and S representing α helices and β strands, respectively. The secondary structure elements characteristic of PAS domains have been numbered from A to I. The arrows indicate the borders of the recombinant proteins (see text). The residues modified by site-directed mutagenesis are marked by asterisks and numbered. The C-terminal part of the sequence comprises the dimerization helix of the kinase (DHp) including the phosphorylated His (highlighted). The numbering starts at the initiation Met of BvgS.

In order to study its function in BvgS and perform its biochemical characterization, we produced PAS_Bvg_ as a recombinant protein in *E. coli*. The PAS core domain (whose limits are given by the N0 and C0 marks in Figure 1) carrying an N-terminal 6-His tag was insoluble. Thus, we produced longer recombinant proteins that also encompass the N- and C-terminal extensions flanking the PAS core and predicted to form α helices (marked NL and CL in Figure 1), as fusions either with an N-terminal 6-His tag or an N-terminal GB1 domain. Because the first protein was totally insoluble and the second was soluble and monomeric, we suspected that the latter might be partly misfolded but protected from aggregation by the GB1 domain, which is known to enhance solubility of its fusion partner [18]. Therefore, we used a more systematic approach by designing several constructs of varying lengths (marked N1, N2, N3, C1, C2 and C3 in Figure 1), and we expressed them under the control of the tightly regulated *tet* promoter. Among these proteins, only N2C2, N2C3, N3C2 and N3C3 were produced in good amounts in essentially soluble forms and could be purified. Size-exclusion chromatography indicated the exclusive formation of dimers for all four of them (not shown). Denaturation of the recombinant proteins using a thermal shift assay (TSA) [23] indicated melting temperatures (Tm) of 61-70°C, arguing that they are properly folded (Table 1). N2C2 and N2C3 had the highest denaturation temperatures. Both contain relatively long extensions on both sides of the PAS core (Figure 1). The reason why the N1 constructs were poorly soluble is unclear. Inspection of the sequence shows the presence of several positively charged residues at their N terminus, leading us to speculate that dimer formation might be destabilized by electrostatic repulsion between the N termini of the two chains.

**Table 1:** Relevant features of the proteins produced in this work

**Name**	Residue range*	**Calculated MW (Da)**^**#**^	**Tm (°C)**
PAS core	592-697	13,193	nd
N1C1	566-701	16,960	nd
N1C2	566-714	18,438	nd
N1C3	566-720	19,049	nd
N2C1	573-701	15,994	nd
N2C2	573-714	17,472	69.7 ± 0.2
N2C3	573-720	18,083	70.5 ± 0.3
N3C1	581-701	15,096	nd
N3C2	581-714	16,574	63.1 ± 0.2
N3C3	581-720	17,185	61.1 ± 0.5
Y_596_A + N_631_A	573-720	17,948	nd
C_607_A	573-720	18,051	62.3 ± 0.2
N_608_A	573-720	18,040	nd
N_608_S	573-720	18,056	60.3 ± 0.6
H_643_A	573-720	18,017	63.0 ± 0.4
R_670_A	573-720	17,998	66.8 ± 0.2
D_695_A	573-720	18,039	60.1 ± 0.1

* The numbering refers to full-length BvgS starting from the initiation Met residue.

# The calculated molecular masses (for a monomer) comprise the start linker from the pASK plasmid without the initiation Met (ASRGSHHHHHHGA). For the PAS core the start linker sequence is RGSHHHHHHGS.

nd, not determined (see text).

The Tms of the N2C3 variants were all significantly different (P < 0.01) from that of the wt N2C3 protein.

Thus, recombinant PAS_Bvg_ produced in *E. coli* is dimeric, and the flanking helices predicted to form coils that precede and follow the PAS core appear to stabilize it. Most kinases of two–component systems work as dimers, and therefore the finding that the domain immediately preceding the kinase in BvgS also dimerises is not unexpected. In addition, PAS domains of other proteins frequently form dimers. It is thus likely that PAS_Bvg_ dimerises in the context of the full-length protein as well.

### PAS_Bvg_ structural model

We next attempted to obtain the X-ray structure of recombinant PAS_Bvg_. However, none of the four soluble recombinant proteins yielded diffracting crystals in spite of repeated attempts. We therefore searched for a homolog of known structure in the protein structure database, on the basis of which a 3-dimensional model of PAS_Bvg_ could be built. The closest PAS domain of known structure, PAS_Hm_ (pdb code: 3BWL), found in an Htr-like protein of *Haloarcula marismortui* has been crystallized in a structural genomic program. PAS_Hm_ is dimeric and harbors a long α helix N terminal to the PAS core, like that predicted to link the transmembrane segment and PAS core domain in BvgS. Interestingly, the physico-chemical properties of these N-terminal flanking α helices are very similar between PAS_Hm_ and PAS_Bvg_, with a number of charged residues in both cases. In the full-length protein of *H. marismortui*, the PAS_Hm_ domain is followed by a predicted α helix and a histidine-kinase domain, like in BvgS. However, PAS_Hm_ was crystallized without this C-terminal α helix. The features of PAS_Hm_ – dimerisation and the presence of flanking helical extensions at both extremities are in agreement with the predictions and available data for PAS_Bvg_, indicating that the former represents a reasonable structural template for the latter.

**Figure 2:**
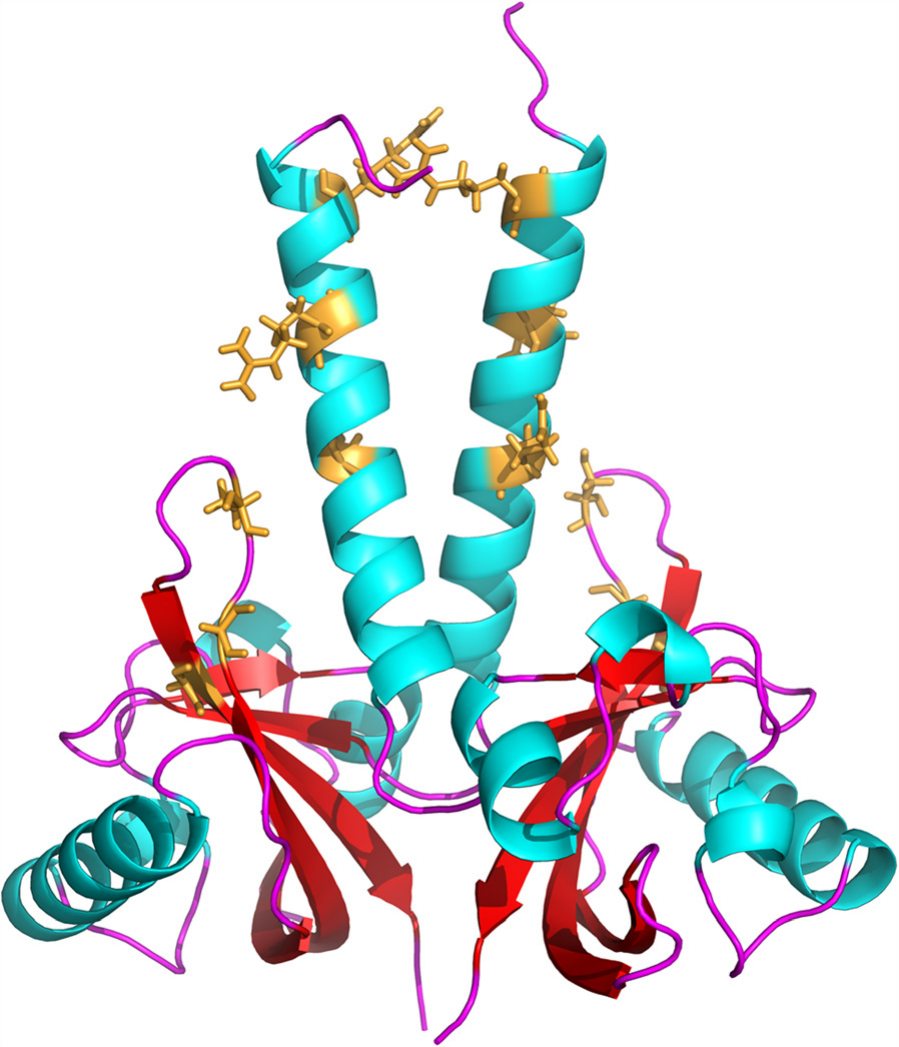
**Structural model for PAS**_**Bvg**_**.** The modeled sequence encompasses residues 564–697 of BvgS, thus immediately following the predicted transmembrane segment of BvgS. The segment after the PAS core has not been modeled, because the corresponding segment is absent from the PAS_Hm_ X-ray structure. In BvgS this segment is predicted to form an α helix linking the PAS and kinase domains. In yellow are shown residues whose substitutions were previously reported to abolish the responsiveness of BvgS to negative modulation (see discussion).

A structural model of PAS_Bvg_ was thus built *in silico* (Figure 2). According to this model, two monomers form a parallel dimer, with long N-terminal, amphipathic α helices extending upward from the PAS cores. Each PAS_Bvg_ core domain is flanked by the last part of the flanking N-terminal α helix of the opposite monomer, thereby forming a swapped dimer. Interactions between these long α helices and between the PAS domains themselves through the backs of their β sheets also contribute to the dimeric interface.

### Hypothesis of a heme co-factor

**Figure 3:**
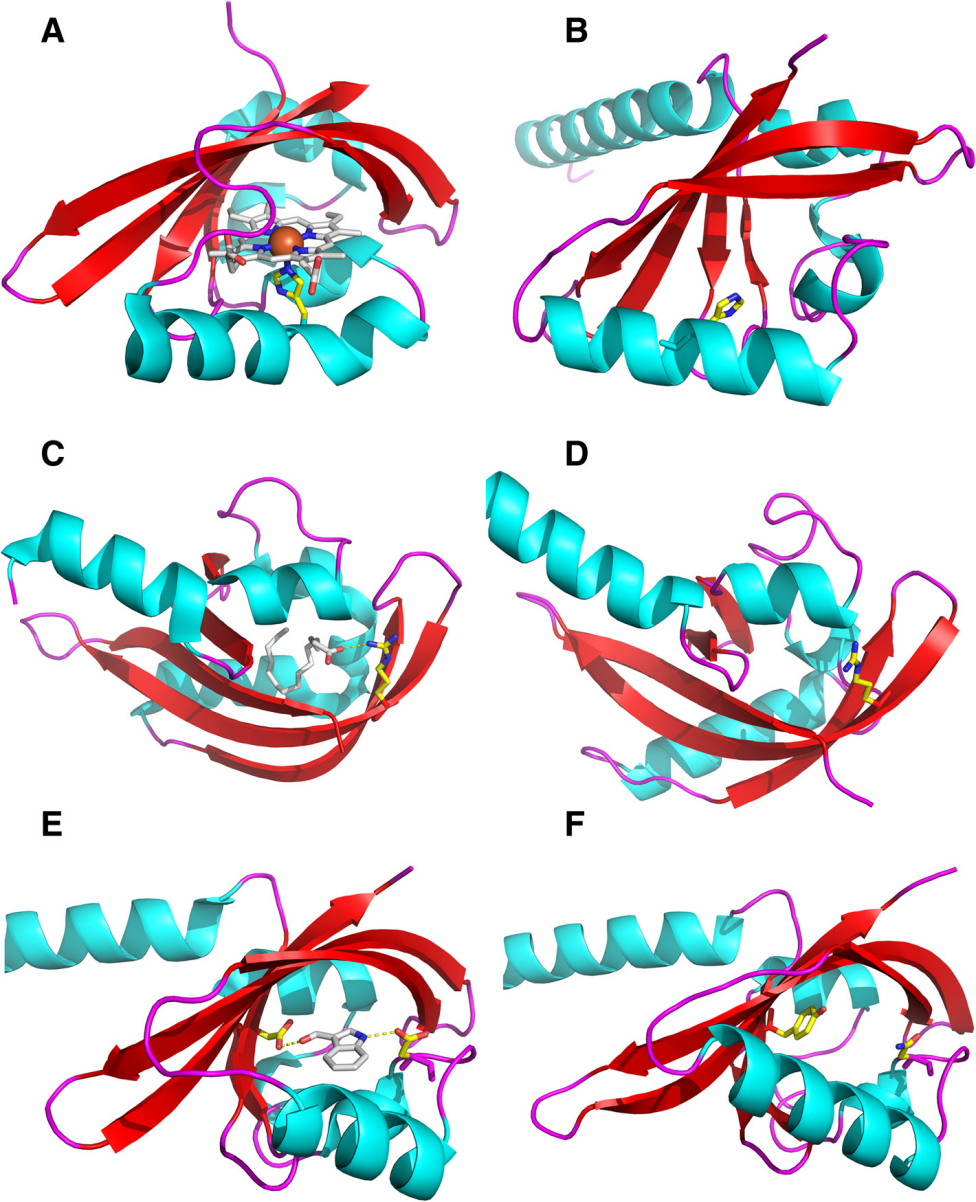
**Close-up views of regions targeted by site-directed mutagenesis.** The structures of PAS domains used to select the residues to replace are shown on the left **(A**,**C**,**E)**, and the corresponding views of the PAS_Bvg_ model are shown on the right **(B**,**D**,**F)**. The residues that were replaced are colored yellow. **A**, FixL_Bj_ PAS domain (pdb code: 1DRM), with the heme colored grey. **C**, PAS domain of the *M. tuberculosis* Rv1364c protein (pdb code: 3KC3), showing the fatty acid in the cavity (in grey). **E**, cavity of PAS_Hm_ (pdb code: 3BWL) with the Asp side chains (in yellow) pointing to the 1H-indole-3 carbaldehyde ligand (in grey). In PAS_Bvg_**(F)** the corresponding residues are Tyr_596_ and Asn_631_.

PAS_Bvg_ shares sequence similarity, and in particular a conserved His residue, with heme-PAS domains of the O_2_-sensing FixL proteins of *Bradirhizobium japonicum* and *Sinorhizobium meliloti*[29-31]. In FixL this His residue serves as an axial ligand for the heme iron. In the PAS_Bvg_ model, the corresponding His residue (His_643_) is located in the long α helix F, with its side chain pointing to the cavity in an appropriate position to interact with a putative heme co-factor (Figure 3). However, the absorbance spectrum of the recombinant PAS_Bvg_ proteins did not indicate the presence of a heme moiety and was not modified by the addition of heme after purification (not shown). Furthermore, when production of PAS_Bvg_ was performed with the addition of hemin or the heme precursor levulinate to the growth medium, no absorbance peak indicative of a heme protein was observed for the purified protein.

**Figure 4:**
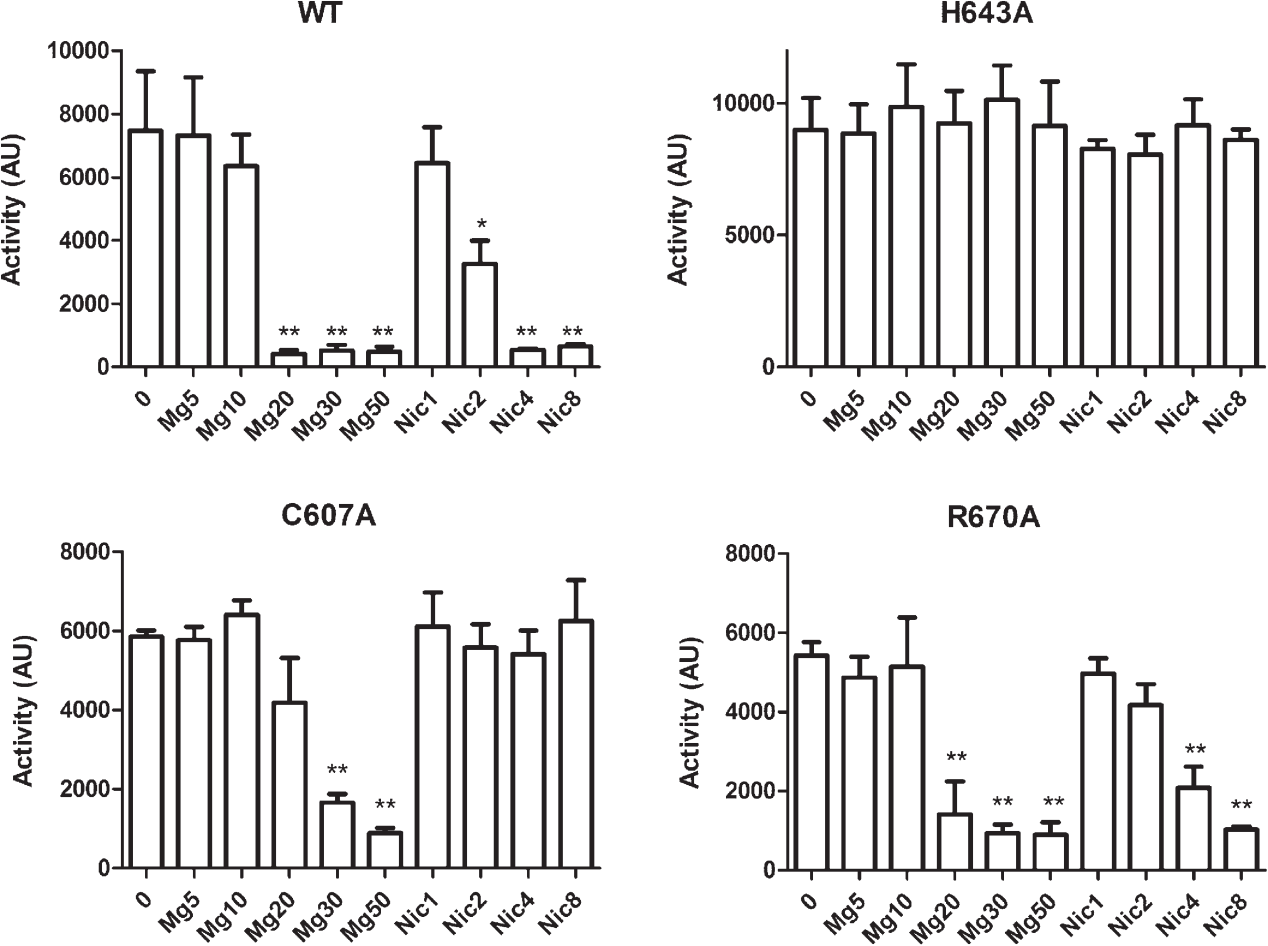
**β-galactosidase activities of the recombinant strains producing the BvgS variants.** The β-galactosidase activities of the *ptx: lacZ* fusion were measured as a function of increasing concentrations of nicotinate or MgSO_4_. The basal (non-modulated) activities of the three variants tested were not significantly different (P > 0.1) from that of wild type (WT) BvgS. The BPSMΔ*bvgS* and BPSMΔ*bvgA* variants had hardly detectable levels of β-galactosidase activities in all conditions, and therefore they were not included in the figure. In each panel, one and two asterisks represent significantly different activities (with P < 0.05 and P < 0.01, respectively) than that of the same non-modulated BvgS variant.

We nevertheless tested the possibility that PAS_Bvg_ harbors a heme co-factor or a related molecule when present in the full-length BvgS protein in *B. pertussis* by replacing His_643_ with Ala. In *bona fide* heme-PAS domains, replacement of the His residue abolishes heme binding [31]. Because *B. pertussis* is virulent in aerobic growth conditions, we reasoned that O_2_ would most likely be a positive signal for BvgS if the PAS domain harbored an O_2_-sensing heme, and therefore that a substitution abolishing heme binding should inactivate BvgS. The mutation was introduced into the chromosome of the *B. pertussis Tohama* I derivative BPSM_E705_ by allelic exchange, and the activity of BvgAS was assessed by using a *lacZ* reporter under the control of the *ptx* promoter, which is positively controlled by BvgAS. The mutated strain expressed ß-galactosidase activity at a level similar to that of the strain containing wt BvgS (Figure 4). Interestingly, BvgS_His643Ala_ was insensitive to sulfate and nicotinate (Figure 4). Other negative modulators [32] also failed to modulate the activity of the recombinant strain, even at much higher concentrations than those that modulate wild type BvgS (not shown). Thus, the His_643_Ala substitution appears to make BvgS unresponsive to modulation.

The His_643_Ala substitution was also introduced into the N2C3 recombinant protein, and the N2C3 variant was purified. Similar to all soluble proteins produced in this work, N2C3_His643Ala_ was dimeric (not shown). Using the thermal shift assay its Tm was determined to be 7°C lower than its wt counterpart (Table 1). Altogether, our data do not support the notion that PAS_Bvg_ has a heme cofactor. However, His_643_ appears to be required for BvgS response to negative signals, indicating its functional importance. It also contributes to the thermal stability of recombinant PAS_Bvg_.

### Role of the PAS cavity

To probe for the presence of another potential co-factor or ligand in the PAS_Bvg_ cavity, electron spray ionization mass spectrometry was performed in native conditions using the N2C3 variant. This analysis independently confirmed the dimeric nature of the recombinant protein, with a mass of 36,171 ± 3.6 Da, and ruled out the presence of a covalent ligand associated with recombinant PAS_Bvg_. A mass spectrometry analysis performed under denaturing conditions yielded a mass of 18,084 ± 1.8 Da, close to the calculated value (18.083 kDa excluding the initiation methionine).

We then targeted other residues of the PAS_Bvg_ cavity between the inner surface of the β sheet and the helices of the PAS core. These residues were chosen on the basis of the structural model and of sequence alignments. PAS_Bvg_ harbours a unique Cys residue (Cys_607_) in a short loop bordering the cavity. Cys residues have been implicated in co-factor binding in other types of PAS (e.g. LOV domains) [33]. In addition, they may be involved in the perception of redox signals [34], a function that has been proposed for BvgS [15]. The substitution of Cys_607_ by an Ala residue in full-length BvgS did not modify its basal activity in *B. pertussis* (Figure 4). Interestingly, BvgS_Cys607Ala_ was non-responsive to modulation by nicotinate, whereas it remained responsive to modulation by MgSO_4_. The responses to other modulators related to nicotinic acid were also tested (not shown). The activity of BvgS_Cys607Ala_ was modulated only at much higher modulator concentrations than those required for the wild type control, indicating that this variant has an intermediate rather than a non-responsive modulation phenotype.

The corresponding recombinant protein was produced, purified and analyzed by TSA. Its Tm was 8°C lower than that of wt N2C3 (Table 1). Altogether, these results identified a second residue of the PAS_Bvg_ cavity whose replacement decreases both the denaturation temperature of the recombinant protein and the ability of BvgS to respond to nicotinic acid and related molecules that are perceived by the periplasmic domain.

The structure of the PAS domain of the *Mycobacterium tuberculosis* Rv1364c protein (pdb code 3K3C) shows an Arg residue in the cavity that is essential for the binding of a C16-fatty acid ligand [22]. An Arg residue is found in PAS_Bvg_ at a corresponding position (Arg_670_), and its side chain appears to be oriented in the same manner in the PAS_Bvg_ model as that in PAS_Rv1364c_ (Figure 3). In the latter protein, the ligand was identified only when the recombinant bacteria were grown at low temperatures (16°C) [22]. We therefore purified N2C3 from *E. coli* grown at 16°C and subjected it to thermal shift analysis before and after delipidation, to test whether the loss of a putative ligand might destabilize the PAS_Bvg_ domain. However, the Tm of N2C3 was not affected by this treatment, and it was similar to that measured for the protein grown at 37°C (not shown). Similarly, the Tm of the N2C3 protein harboring the Arg_670_Ala substitution was only moderately decreased compared to that of its wt counterpart (Table 1). We also introduced the Arg_670_Ala substitution in full-length BvgS, which did not affect its activity in *B. pertussis* or its ability to respond to negative signals (Figure 4). These observations thus rule out a major function for this residue in PAS_Bvg_.

**Figure 5:**
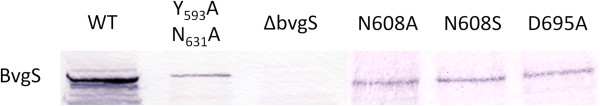
**Detection of inactive BvgS variants in membrane extracts of the recombinant *B. pertussis *strains.** The immunoblots were revealed using anti-BvgS polyclonal antibodies. The one-letter code was used to denote the substitutions. Δ*bvgS* represents BPSMΔ*bvgS* from which *bvgS* has been deleted.

More drastic changes in the PAS cavity were next engineered. In the 3BWL structure, the side chains of two Asp residues bind a fortuitously trapped 1H-indole-3 carbaldehyde ligand in the PAS cavity. The side chains of the residues at those positions are frequently involved in ligand binding by other PAS domains (our observations), and in the PAS_Bvg_ cavity these positions are occupied by Tyr_596_ and Asn_631_ (Figure 3). They were replaced together by Ala in full-length BvgS. BvgS in the resulting *B. pertussis* recombinant strain was totally inactive (not shown). We thus verified that BvgS_Tyr596Ala+Asn631Ala_ was produced in a stable form in the recombinant *B. pertussis* strain by preparing membrane extracts and subjecting them to immuno-blotting using polyclonal anti-BvgS antibodies (Figure 5). The protein was detected, showing that the substitutions did not disrupt full-length BvgS or cause its proteolytic degradation but affected its function.

We next determined the effect of the Tyr_596_Ala + Asn_631_Ala substitutions on the thermal stability of the recombinant protein. Surprisingly, although N2C3_Tyr596Ala+Asn631Ala_ was purified in a soluble and dimeric form in good amounts, no cooperative denaturation profile was obtained by TSA, and thus no Tm could be calculated. This suggested a significantly looser structure of the PAS core even at lower temperatures. The observations that the joint replacements of Tyr_596_ and Asn_631_ in the PAS_Bvg_ cavity both abolished BvgS activity and considerably destabilized PAS_Bvg_ argue that the structural stability of the PAS core domain is important for BvgS function. Of note, mutations in the PAS core have been shown to affect the stability and function of other PAS domains as well [35,36].

### PAS coupling with flanking regions

**Figure 6:**
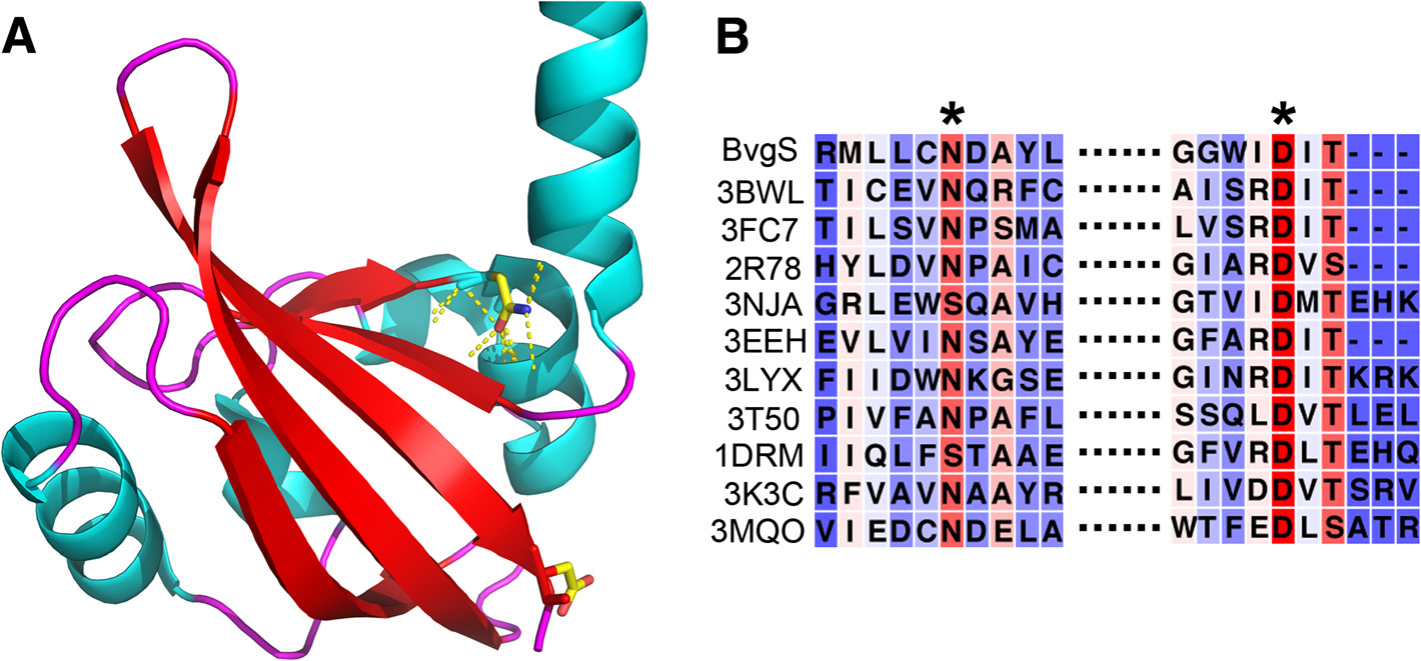
**View of the connection between the PAS core and the flanking N-terminal α helix in the PAS**_**Bvg **_**model. A**, The hydrogen bonds between the conserved Asn residue and the PAS core and N-terminal α helix are shown in stippled lines. Because the C-terminal α helix is absent from the model, the connections of the conserved Asp residue with the flanking C-terminal α helix could not be represented. **B**, Sequence alignments of these conserved regions are shown in two blocks on the right-hand side of the figure, with the pdb code numbers of the PAS proteins used for the alignment. The conserved Asn/Ser and Asp residues are denoted with asterisks.

Based on those results, we hypothesized that a major function of the PAS domain is to maintain - and perhaps to amplify- conformational signals coming from the periplasmic moiety of BvgS to the kinase domain, thus requiring a tightly folded PAS core properly connected to the upstream and downstream α helices. To test this hypothesis, we modified residues that couple the PAS domain to its flanking helices and determined the effects of these replacements on BvgS activity. In the PAS_Hm_ structure, a highly conserved Asn residue corresponding to Asn_608_ in BvgS forms hydrogen bonds within the PAS core and docks the core to the flanking N-terminal α helix (Figure 6). This conserved Asn residue is critical for signaling in PAS proteins such as PYP of *Halorhodospira halophila* and Aer of *Escherichia coli*[36,37]. In many PAS domains, a conserved D(I/V/L)T motif terminates the PAS core, whose Asp and Thr side chains make interactions that couple it with its flanking C-terminal α helix and effector domain downstream [8,38] (Figure 6). The corresponding Asp residue in PAS_Bvg_ is Asp_695_. To determine the importance of these motifs in BvgS, Asn_608_ and Asp_695_ were separately replaced by Ala in full-length BvgS, and Asn_608_ was also replaced by Ser to maintain some H-bonding capability of the side chain. Of note, a Ser residue is naturally found at this position in certain PAS domains (Figure 6). All three substitutions had dramatic effects on Bvg activity in *B. pertussis*, making the protein inactive in all three cases (not shown). The three variants were nevertheless detected in membrane extracts of the recombinant strains (Figure 5). Thus, the corresponding substitutions abolished the function of BvgS but did not hamper its membrane localization nor cause its degradation.

To determine whether these substitutions affected the PAS_Bvg_ structural integrity, they were introduced into the recombinant N2C3 protein, and the thermal stabilities of the three variants were determined (Table 1). The N2C3_Asn608Ala_ protein was produced in very low amounts, suggesting that the substitution considerably affects its structural integrity. The soluble fraction of the protein was dimeric but had a tendency to precipitate, and therefore it could not be analyzed further. In contrast, the other two proteins were produced in reasonable amounts although lower than that of wt PAS_BvgS_ in soluble, dimeric forms, and they were relatively stable over time, suggesting that they were properly folded. Nevertheless, their Tms were more than 10°C lower than that of the corresponding wt protein (Table 1). Thus, disconnecting the PAS domain from the flanking helices both abolishes BvgS activity and significantly decreases the stability of recombinant PAS_Bvg_. The loss of BvgS activity seems to correlate with significantly looser PAS domain structures.

## Discussion

Over 20,000 PAS domains have been identified in different proteins. They are essentially involved in regulation or sensing. In the family of VFT-containing sensor-kinases of which BvgS is a prototype, PAS domains are frequently found between the transmembrane segment and the kinase domain. Sequences of the *bvgAS* locus from a number of *B. pertussis*, *Bordetella bronchiseptica* and *Bordetella parapertussis* isolates have shown the remarkable conservation of the PAS domain in BvgS, supporting the idea that it is functionally important [19]. In this work, we identified specific amino acid residues in the PAS domain whose substitutions abolish BvgS activity. They map to three different locations: at the interfaces between the PAS core and its flanking N-terminal and C-terminal α helices, and in the PAS cavity. These results support a key transmission function for the PAS domain in BvgS, related to its critical position between the periplasmic and kinase domains. The PAS domain in BvgS needs to be tightly folded to fulfill this role, because significantly loosening the PAS core or its connections with upstream and downstream helices dramatically affects BvgS activity.

We found that the PAS_Bvg_ domain dimerises in *E. coli*, and we propose that it does so in full-length BvgS as well. Dimer formation is consistent with earlier findings that the kinase domain of BvgS dimerises [39-41]. The increased solubility of recombinant PAS_Bvg_ proteins containing large portions of the C- and N-terminal flanking α helices argues that the latter contribute to dimer formation, as described for some other PAS domains [42,43]. The outer surfaces of the β sheet of PAS cores are generally hydrophobic, and in other PAS dimers they participate in the interface or are apposed to flanking helices [8,13,44]. This also appears to be the case for PAS_Bvg_. The structural model is also in good agreement with proposed mechanisms of signal transmission by other PAS domains, with the β sheet participating in signaling [43,45,46]. In the PAS_Bvg_ model the β sheet is well positioned to relay information to the flanking C-terminal α helix and thus to the kinase domain.

In the current mechanistic model, BvgS is active in its basal state, and this activity requires the integrity of the periplasmic domain, since specific substitutions or insertions in the periplasmic region of BvgS abolish activity [6,47]. We thus propose that in its basal, non-liganded state the periplasmic domain adopts a conformation that provides a positive signal to the system. The binding of nicotinate to the VFT2 domain modifies this conformation and strongly decreases the positive-signaling capability of the protein [6]. The distinct conformational states of the periplasmic domain most likely impose distinct conformations onto the membrane segment that are propagated via long α helices to the PAS_Bvg_ domain and from there to the kinase domain underneath. In this work, we found that disruption of the coupling between PAS_Bvg_ and its upstream or downstream α helices abolishes BvgS activity, as do some of the PAS cavity substitutions. We therefore propose that the conformation of the periplasmic domain generates mechanical strain in BvgS, and that a major function of the PAS domain in BvgS is to maintain, and possibly to amplify, this conformational signal. The complete loss of activity of some BvgS variants generated in this study correlates with strong decreases in thermal stability of the recombinant PAS domain. The corresponding substitutions thus cause considerably looser structures that most likely make the PAS_Bvg_ domain unable to maintain and/or transmit the proper conformational strain to the kinase. The importance of the PAS core for stability and activity has also been shown for other PAS domains [35,36].

Another observation from this and previous work is that a number of substitutions in the PAS domain do not inactivate BvgS but render it unresponsive to negative modulation by nicotinate and sulfate [16,47,48]. Previously reported substitutions that make BvgS unresponsive to modulation map essentially to a PAS core loop oriented towards the N-terminal flanking helix or to the N-terminal helix itself (Figure 2). It is thus likely that they affect the connection between the PAS core and the upstream region or the stability of the PAS dimer through its N-terminal helices. In the current work, new substitutions that impair or abolish responsiveness to modulation were also identified in the PAS cavity. The structural stabilities of the latter two PAS_Bvg_ variant proteins appeared to be decreased to a lower extent than those of the inactive proteins. The observation that the unresponsive BvgS PAS variants remain competent to transmit positive but not negative signals suggests that transmission of modulating signals implies an increased conformational strain relative to the basal, positive-signaling state.

Our results do not support the hypothesis that PAS_BvgS_ has a heme co-factor. Thus, the His_643_Ala substitution does not abolish BvgS activity, as would be expected from the loss of an O_2_-sensing heme for a strictly aerobic and virulent bacterium. However, this substitution abolishes the response of BvgS to negative modulation, and another substitution in the PAS cavity (Cys_607_Ala) also decreases BvgS sensitivity to nicotinate. These effects might be explained either by a moderate loosening of the PAS core because the small Ala side chain replaces a larger one, which disrupts the transmission of negative signals, or by a defect in binding a potential intracellular ligand required for transmission of negative signals. The double Tyr_596_Ala + Asn_631_Ala substitutions in the PAS cavity that abolish BvgS activity and strongly decrease the PAS thermal stability might also disable ligand binding *in vivo*. Binding of a cytoplamic ligand by the PAS domain would be consistent with the established link between the nutritional state of *B. pertussis* and its virulence [49] and with the observations that BvgAS regulates cytochrome expression and might perceive redox signals [17,50]. Thus, the PAS_Bvg_ domain might sense intracellular molecule(s) whose abundance reflect(s) the metabolic state of the bacterium, and changes to the concentration of these components might affect signaling. Such a scenario would be compatible with the ‘rheostat’ behavior attributed to BvgS [3]. In any case, the effects of cavity mutations on BvgS activity lend strong support to our model that the conformation of the PAS core –intrinsically or by virtue of ligand binding- is critical for signaling.

## Conclusions

Although substantial information has been gathered about how the cytoplasmic domains of BvgS work, the function of its PAS domain has remained unknown. In this work, we performed its characterization, which represents new information that contributes to our understanding of VFT-containing sensor-kinases. We showed that the recombinant PAS domain of the sensor-kinase BvgS dimerises, and that the N- and C-terminal α-helical regions that flank the PAS core are critical for dimer stabilization. We identified specific amino acid residues in the PAS domain that are essential for BvgS activity, located in the PAS core and at the junctions between it and its flanking α helices. We thus propose a mechanical role for the PAS domain in BvgS, which is to maintain the conformational tension imposed by the periplasmic moiety of BvgS. The degree of tension in the protein determines the activity of the kinase, and modulation corresponds to an increased tension. Our model thus explains for the first time the phenotypes of a number of BvgS variants that harbor mild substitutions in the PAS domain and are unable to respond to negative modulation.

## Abbreviations

TCS: Two-component system; PAS: Per/ARNT/Sim; VFT: Venus flytrap domain; TSA: Thermal shift assay; Tm: Melting (denaturation) temperature

## Competing interests

The authors declare no competing interests.

## Authors’ contributions

ED, JH, FJ-D and RA designed the study; ED, JH, AW performed the experiments; ED, JH, AW, CL, FJ-D and RA wrote the paper. All authors have read and approved the final version of manuscript.

## Supplementary Material

Additional file 1: Table S1 Oligonucleotides used in this study. Click here for file
