# Supplementary material for: Characterization of the PAS domain in the sensor-kinase BvgS: mechanical role in signal transmission
Source: BMC Microbiol. 2013 Jul 24;13:172. doi: 10.1186/1471-2180-13-172 (PMC3726324; doi:10.1186/1471-2180-13-172)
Supplement: Additional file 1: Table S1 — Oligonucleotides used in this study. [file 1471-2180-13-172-S1.pdf]

Additional file 1.

Table S1. Oligonucleotides used in this study.

PAScore LO: 5'-ATAAGCTTCTAGGTGATGTCGATCCAGCC-3'

PAScore UP: 5'-AAGGATCCCCTAACCCCATCTATGTGCG-3'

PAS His UP: 5'-ATAGATCTCGACAGATCCGCCAGCGCAAGC-3'

PAS His LO: 5'-ATAAGCTTTGCCAGGAACGTGGTCTTGG-3'

PAS GB1 UP: 5'-ATGGATCCCGACAGATCCGCCAGCGCAAGC-3'

PAS GB1 LO: 5'-ATCTCGAGTGCCAGGAACGTGGTCTTGG-3'

PAS N1UP: 5'-ATGGTAGGTCTCAGCGCCCAGATCCGACAACGCAAGCG-3'

PAS N2UP: 5'-ATGGTAGGTCTCAGCGCCGCCGAACGGGCACTGAACG-3'

PAS N3UP: 5'-ATGGTAGGTCTCAGCGCCCTGGAATTCATGCGAGTGCTCA-3'

PAS C1LO: 5'-ATGGTAGGTCTCATATCACTCGGCGCGTTCGGTGATGTC-3'

PAS C2LO: 5'-ATGGTAGGTCTCATATCAGTCGGCACTTTCCTTGGCGTC-3'

PAS C3LO: 5'-ATGGTAGGTCTCATATCACTTGGCTCGGTTGGCGGCG-3'

Bvg-BglII UP: 5'-ACCGCAACGAGATCTACGTG-3'

Bvg-XbaI LO: 5'-TATCTAGAATGTCGCCTATCAGC-3'

BvgY_{596}A UP: 5'-GGCACGCCTAACCCCATAGCTGTGCGCGATAAGGAAGGCCG-3'

BvgY_{596}A LO: 5'-AGCTATGGGGTTAGGCGTGCC-3'

BvgC_{607}A-UP 5'-GAAGGCCGCATGCTGTTGGCTAATGACGCCTACCTCGACAC-3'

BvgC_{607}A-LO 5'-AGCCAACAGCATGCGGCCTTC-3'

BvgN_{608}A-UP 5'-AGGCCGCATGCTGTTGTGTGCTGACGCCTACCTCGACACCT-3'

BvgN_{608}A-LO 5'-AGCACACAACAGCATGCGGCCT-3'

BvgN_{608}S-UP 5'-AGGCCGCATGCTGTTGTGCTCTGACGCCTACCTCGACACCT-3'

BvgN_{608}S-LO 5'-AGAGCACAACAGCATGCGGCCT-3'

BvgN_{631}A UP: 5'-AAACCATTCCGGAAGCTGCAGTGGTGGGCGACCCGGCGCTG-3'

BvgN_{631}A LO: 5'-TGCAGCTTCCGGAATGGTCTTGC-3'

BvgH_{643}A UP: 5'-GCGCTGGCTCGAGAAATGGCTGAGTTCCTGCTCACGCGCGTG-3'

BvgH_{643}A LO: 5'-AGCCATTTCTCGAGCCAGCGCCGGGTCG-3'

BvgR_{670}A-UP 5'-CACGCTGCACGGCCGCACGGCTCATGTCTACCAGTGGACGAT-3'

$BvgR_{670}A$-LO 5'-AGCCGTGCGGCCGTGCAGCGTG-3'

$BvgD_{695}A$-UP 5'-ATCATCGGAGGCTGGATTGCTATCACCGAACGCGCCGAGCTG-3'

$BvgD_{695}A$-LO 5'-AGCAATCCAGCCTCCGATGATGCCCTTGAGT-3'

BvgA-UP1 5’-ATTCTAGATGAAATCCAGTGCCATAGTCT-3’

BvgA-LO1 5’-TACAGGGTGATCGTCAATGATG-3’

BvgA-UP2 5’-CATCATTGACGATCACCCTGTAAAACGCAACAATCTCGCCTAGC-3’

BvgA-LO2 5’-ATAAGCTTGCCATTGACGGTGCCGATGAG-3’
